# The 4^th^ National Neurology Forum | Interview with Prof. Dr Dafin Fior Mureșanu

**DOI:** 10.25122/jml-2026-1009

**Published:** 2026-05

**Authors:** Stefana-Andrada Dobran, Alexandra Gherman

**Affiliations:** 1 RoNeuro Institute for Neurological Research and Diagnostic, Cluj-Napoca, Romania

**Interviewee: Prof. Dr Dafin Fior Mureșanu**

**Interviewer: Stefana-Andrada Dobran**

**S.D.: Hello, Professor Dr Dafin Fior Mureșanu, and welcome to the fourth edition of the National Neurology Forum in Bucharest, Romania. What do you consider to differentiate this forum from other scientific events in the field?**

D.F.M.: Thank you very much. As the organiser of this event and this interaction model, I would say that the forum represents a model of engagement between professional fields and different social segments, each with distinct functions. It is not dedicated exclusively to science, so it is not a purely scientific event, but rather an integrative working model for areas of interest that have their own “metabolism” and independent dynamics, and which very often do not communicate with each other.

The forum is important because it creates this framework: a quadrilateral formed by medical professionals, patients – who are extremely important –, the authorities that regulate elements related to the doctor-patient interaction, alongside industry and other stakeholders. Therefore, this is the place where all the mentioned stakeholders come together.

I do not think that, up to this point, there is a better framework that brings together and highlights the issues faced by each party. Very often, there are asymmetries in society and daily life. What do these asymmetries lead to? Conflicts. I do not mean open conflicts. Conflict also means a lack of mutual understanding and an accumulation of frustrations and negative energies that are ultimately unproductive.


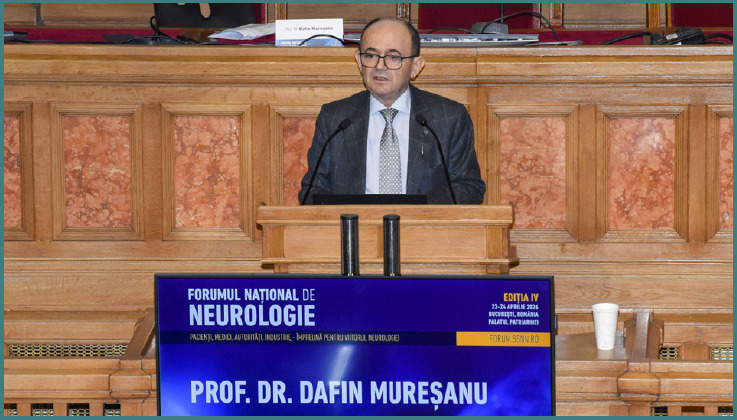


This is what sets apart the National Neurology Forum, for example, from the National Neurology Congress, where the interaction is exclusively scientific and medical.

**S.D.: What are the main objectives of the National Strategy for Combating Cardiovascular and Cerebrovascular Diseases 2025-2030, and how can this strategy contribute to improving the prevention, diagnosis and treatment of cardio- and cerebrovascular diseases in Romania?**

D.F.M.: You have already mentioned part of the meaning of the strategy. You referred to prevention, which is fundamental, and to treatment, which is equally important. Ultimately, any strategy must have the patient as its central element.

How can the approach to stroke care be improved from the patient’s perspective? It is both simple and complex, in fact. Simple, because all these modules, practised by different specialities, must be properly integrated. In essence, it is a patient pathway that depends on multiple specialities, which must work together to form a coherent whole and a functional system.

When we think about prevention, we first need to understand the nature of the risk. There must a well-defined epidemiological perspective. So, we are talking about epidemiologists– professionals with deep, operational understanding of the health systems.

In the pre-hospital phase, we have significant challenges related to population behaviour toward this pathology. In stroke, recognition is critical: the earlier it is identified, the better the outcome. Unlike conditions such as Parkinson’s disease—where delaying the consultation for a week may not significantly change the course of care—in stroke, we are dealing with minutes and hours. Therefore, it is of utmost importance to recognize symptoms early and address them immediately.

A pre-hospital system must activate the emergency chain that takes the patient to the right place, at the right time, and to the right people. Any misalignment in this chain can lead to exactly the opposite outcome.

Then, coordinating all these elements – understanding and prevention of risk factors – is essential. Raising public awareness is achieved through patient societies, with the support of physicians and scientific societies.

Equally important is the coordination between ambulance services and the hospital emergency system. It is crucial to prioritise patients for whom time is a critical factor, such as those with stroke.

This is followed by in-hospital management, from admission through medical investigations that establish the diagnosis and the decision of whether the patient is eligible for a specific intervention. What needs to be done? All of this has to function in a unitary, coordinated system.

We have some parameters that need to be continuously optimised, such as the “door-to-needle” time. In addition, there are other critical intervals: from the recognition of symptoms to calling the ambulance, and from ambulance arrival to hospital admission. This is where "door-to-needle" begins. But ultimately, the times that matter are the ones that mark the entire journey, from the onset of the accident and symptoms to the therapeutic decision and intervention. All of this has to function in a unitary, coordinated system.


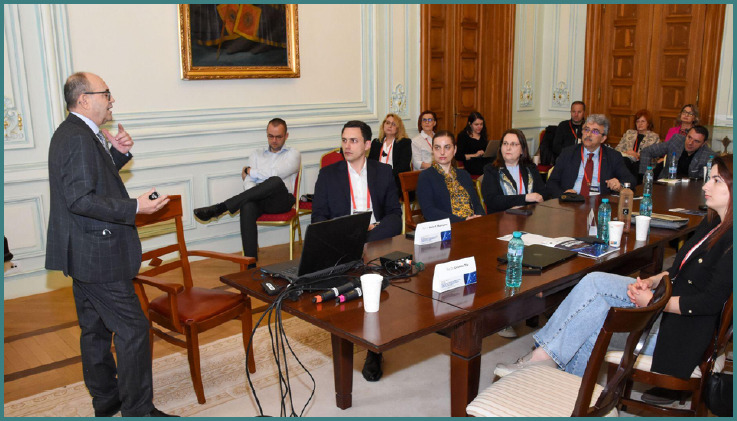


Beyond the acute phase, we must ask what happens to patients after they have been successfully treated. How many patients receive treatment? How many benefit from thrombolysis and/or thrombectomy? What happens to those who do not benefit from these procedures? How does the healthcare system manage them? What happens to each category in the medium and long term? Do they enter a recovery process, or not? Ideally, they should enter a recovery pathway. But what happens to patients when they return? People are different: some have strong family support, while others are alone and in distress. Some understand what is happening to them, others do not. This results in a high degree of heterogeneity in contexts and needs, all of which must be addressed in a coherent system.

All these links must be connected. Each link must understand what is happening with any of the other links. Without a clear strategy across the entire pathway, we are left with a fragmented picture, and tensions between specialities inevitably arise. If, for example, a neuroradiologist has a different perspective from a neurologist and there is no clear regulatory framework, the system can become blocked.

Attitude also needs to be shaped. Attitude is very important and can be shaped both through planned measures and spontaneously through societal culture. For example, in Germany, there is a framework stating that if a person witnesses a life-threatening situation – such as someone collapsing – and they do not call for help and intervene to solve that problem, they may be held legally accountable. In other places, people simply walk past and look away.

All this must be addressed systematically. Where awareness is lacking, it must be strengthened through responsibility. Where there is insufficient collaboration between specialities, this collaboration must be encouraged through a range of measures, from incentives to strict regulation.

Therefore, this is the essence of a strategy. All of these elements must be brought together into a coherent framework–a clear legislative framework, that can be invoked whenever needed, and that defines responsibilities, workflows, staffing, and the way all components function together.


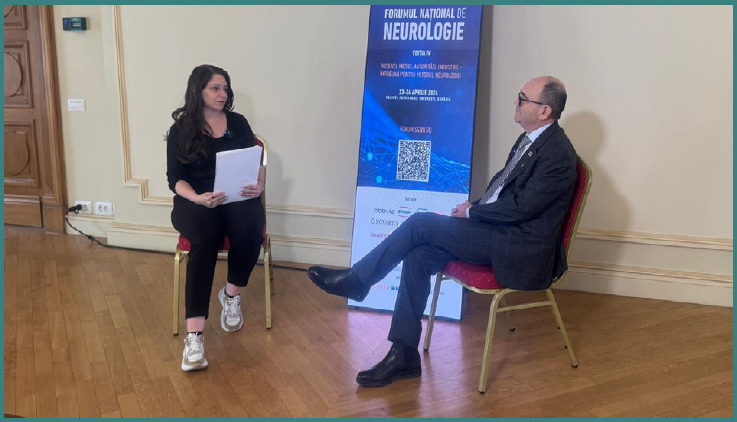


**S.D.: From your perspective, what are the most important directions of development in neurology in terms of research, awareness, and recovery?**

D.F.M.: Of course, the question is very complex. For example, we have patient awareness regarding their own health within the general population. This is an important area, but as a major field of interest in research, I believe that the increasing use of technology to understand brain function is currently the most important direction.

At present, significant resources are being invested in technological approaches aimed at achieving a deep understanding of brain function. Once you understand how the brain works based on this information, data, and knowledge, you can do more things. That is what happens. It can be used to develop interventions and therapies for the benefit of patients. Alternatively, it can also be used to replicate these mechanisms and use them for artificial intelligence, opening up different avenues. There are humanistic neurosciences, which aim to solve human problems, and there are technetronic neurosciences, which aim to enhance artificial intelligence and enable it to function at a higher level in this sense.

When it comes to fundamental research aimed at improving health problems, I believe that a particularly important area is neuroprotection and neuro-recovery, or what we call "brain recovery". Understanding these mechanisms addresses many aspects related to the treatment of our patients across various types of conditions. Therefore, I consider this to be one of the most important areas. Research is essential, and it largely depends on how we direct its results and the direction in which we choose to use them. Of course, all directions are active, some more active than others.

When it comes to public awareness, I think it is very important for people to be aware of what may happen to them. If you are not aware, you remain contemplative, and you do not understand. You need to be aware of what might happen to you in order to engage in prevention, or of what has already happened so that you can respond promptly.

For example, awareness of early detection of stroke is essential. There are simple algorithms that can lead to a rapid understanding of what is happening. For example, the FAST algorithm. It tells you to pay attention to F — “Face” —if you notice facial asymmetry. We are talking about a stroke that occurs suddenly. The A comes from “Arm”, meaning whether you have strength in your arm or not. The S comes from “Speech” - whether you have speech disorders or not- and the T stands for “Time”.

All of this, integrated, is not difficult to explain, but someone must communicate it to the public. Of course, patients often receive this information after the acute event, but ideally, they should be informed in advance about how to reduce the risk. That is, in fact, prevention.

It is an extremely important effort, but also a very difficult one, because the public attention is dominated by other types of messages. We have wars, crises, problems, price increases, accidents, and crimes. There is little space left for messages that could help prevent disease. As a result, a predominantly negative form of awareness is constantly growing, which does not effectively support prevention in this direction.

**S.D.: A thought or a message that you would like to convey at the end of this event?**

D.F.M.: First of all, we must do a better job of working together as professionals and breaking down these barriers of thought, of understanding and, ultimately, of small interests or elements that divide us.

There are the familiar arguments: neurologists do not get along well with neurosurgeons, rehabilitation specialists do not get along with neurologists, and no one gets along with anyone. What we are trying to do is to dissolve these barriers and to realise that it is, after all, better to work together.

At least we, with cardiologists and other specialities, now collaborate very well. This is one example. That is what we need to achieve here. At least that, because it is the one thing within our control.

**S.D.: Thank you very much for the interview**

D.F.M.: Thank you, it was a pleasure.

